# Medidas de distanciamento social como fator de proteção contra a COVID-19 no interior do Rio Grande do Sul, Brasil

**DOI:** 10.26633/RPSP.2021.145

**Published:** 2021-11-19

**Authors:** Ana Paula Helfer Schneider, Mari Ângela Gaedke, Janine Koepp, Éboni Marília Reuter, Camilo Darsie, Lia Gonçalves Possuelo, Andréia Rosane de Moura Valim, Marcelo Carneiro, Grupo COVID-VRP

**Affiliations:** 1 Universidade de Santa Cruz do Sul (UNISC), Departamento de Ciências da Vida Santa Cruz do Sul (RS) Brasil Universidade de Santa Cruz do Sul (UNISC), Departamento de Ciências da Vida, Santa Cruz do Sul (RS), Brasil.; 2 Universidade de Santa Cruz do Sul (UNISC), Programa de Pós-Graduação em Promoção da Saúde, Departamento de Ciências da Saúde Santa Cruz do Sul (RS), Brasil Universidade de Santa Cruz do Sul (UNISC), Programa de Pós-Graduação em Promoção da Saúde, Departamento de Ciências da Saúde, Santa Cruz do Sul (RS), Brasil.; 3 Universidade de Santa Cruz do Sul (UNISC), Programa de Pós-Graduação em Educação, Departamento de Ciências, Humanidades e Educação Santa Cruz do Sul (RS), Brasil Universidade de Santa Cruz do Sul (UNISC), Programa de Pós-Graduação em Educação, Departamento de Ciências, Humanidades e Educação, Santa Cruz do Sul (RS), Brasil.; 4 Grupo de pesquisa COVID-Vale do Rio Pardo: Jane Dagmar Pollo Renner, Adilson Ben da Costa, Renato Michel, Ingre Paz, Daiana Klein Weber Carissimi, Suzane Beatriz Frantz Krug, Eliane Carlosso Krummenauer, Rochele Mosmann de Menezes, Clauciane Zell, Bruna Rezende, Caroline Bertelli, Fernanda Iochimns e Léa Vargas Grupo de pesquisa COVID-Vale do Rio Pardo: Jane Dagmar Pollo Renner, Adilson Ben da Costa, Renato Michel, Ingre Paz, Daiana Klein Weber Carissimi, Suzane Beatriz Frantz Krug, Eliane Carlosso Krummenauer, Rochele Mosmann de Menezes, Clauciane Zell, Bruna Rezende, Caroline Bertelli, Fernanda Iochimns e Léa Vargas.

**Keywords:** COVID-19, distanciamiento físico, prevención de enfermedades, estudios seroepidemiológicos, control de enfermedades transmisibles, Brasil, COVID-19, physical distancing, disease prevention, serology, seroepidemiologic studies, communicable disease control, Brazil, COVID-19, distanciamento físico, prevenção de doenças, estudos soroepidemiológicos, controle de doenças transmissíveis, Brasil

## Abstract

**Objetivo.:**

Investigar a soroprevalência de SARS-CoV-2 na região do Vale do Rio Pardo, Rio Grande do Sul, Brasil, e analisar a associação entre soroprevalência e adesão por parte da população às medidas de distanciamento social.

**Método.:**

Este estudo transversal de base populacional compreendeu quatro etapas de coleta domiciliar de dados entre agosto e outubro de 2020. A soroprevalência foi avaliada utilizando teste rápido de anticorpos IgM e IgG. Foram coletados, ainda, dados demográficos, socioeconômicos, clínicos e comportamentais, com aplicação de um questionário de três perguntas sobre adesão a medidas de distanciamento social, com foco no nível de distanciamento social que o entrevistado conseguia praticar, rotina de atividades do entrevistado e circulação de pessoas na casa. A associação entre os dados sociodemográficos e a prática de distanciamento social foi avaliada pelo teste do qui-quadrado para tendência linear e heterogeneidade de proporções, e a associação entre o distanciamento social e a soroprevalência foi avaliada pela regressão de Poisson (intervalo de confiança de 95% [IC95%]; *P* < 0,05).

**Resultados.:**

Dos 4 252 indivíduos testados e entrevistados, 11,8% (IC95%: 10,8; 12,8) não aderiram ao distanciamento social. A prevalência de teste rápido reagente foi de 4,7% entre aqueles que não realizaram distanciamento social e de 1,9% entre aqueles que realizaram distanciamento social (*P* < 0,05). As variáveis sexo masculino, faixa etária de 20 a 59 anos, ensino médio, renda familiar mensal de R$ 3 136,00 a R$ 6 270,00 e morar na zona urbana apresentaram associação com a não adesão ao distanciamento social (*P* < 0,05). A adesão a todas as medidas de distanciamento social foi fator de proteção contra a infecção de SARS-CoV-2 (razão de prevalência: 0,37; IC95%: 0,19; 0,73).

**Conclusões.:**

Os resultados indicam uma redução da soroprevalência pelas medidas de distanciamento social.

## Abstract (en)

**Objective.:**

To estimate the SARS-CoV-2 seroprevalence in a non-metropolitan area (Vale do Rio Pardo) in the state of Rio Grande do Sul, Brazil, and determine the association between seroprevalence and adherence to social distancing measures.

**Method.:**

For the present population-based, cross-sectional study, data were collected in four stages from August to October 2020. SARS-CoV-2 seroprevalence was assessed using an IgG/IgM rapid test. Demographic, socioeconomic, clinical, and behavioral data were also collected, with administration of a three-question survey to determine adherence to social distancing measures with a focus on the level of social distancing practiced by participants, their routine activities, and circulation of people in the home. The association between sociodemographic data and social distancing was assessed using the chi-square test for linear trends in proportions, and the association between social distancing and seroprevalence was assessed using Poisson regression (95% confidence interval [95%CI]; *P*< 0.05).

**Results.:**

Of 4 252 tested and interviewed participants, 11.8% (95%CI: 10.8; 12.8) did not adhere to social distancing measures. The prevalence of a positive rapid test was 4.7% in participants who did not practice social distancing and 1.9% in participants who adhered to social distancing measures (*P*< 0.05). The variables male sex, age 20 to 59 years, having completed high school, monthly family income ranging from R$ 3 136.00 to R$ 6 270.00, and living in rural areas were associated with non-adherence to social distancing (*P*< 0.05). Adherence to all social distancing measures provided protection against SARS-CoV-2 infection (prevalence ratio: 0.37; 95%CI: 0.19; 0.73).

**Conclusions.:**

The results indicate a reduction in seroprevalence with the adherence to social distancing measures.

## RESUMEN

**Objetivo.:**

Investigar la seroprevalencia del SARS-CoV-2 en la zona del Vale do Rio Pardo (Rio Grande do Sul, Brasil), y analizar la relación entre la seroprevalencia y el cumplimiento de las medidas de distanciamiento social por parte de la población.

**Método.:**

Este estudio transversal basado en la población comprendió cuatro etapas de recopilación domiciliaria de datos entre agosto y octubre del 2020. La seroprevalencia se evaluó con la prueba rápida de anticuerpos IgM e IgG. Además, se recopilaron datos demográficos, socioeconómicos, clínicos y comportamentales por medio de un cuestionario de tres preguntas sobre el cumplimiento de las medidas de distanciamiento social, centrado en el grado de distanciamiento social que la persona entrevistada lograba tener, la rutina de las actividades de la persona entrevistada y la circulación de personas en el hogar. La relación entre los datos sociodemográficos y la práctica de distanciamiento social se evaluó con la prueba del ji cuadrado para determinar la tendencia lineal y la heterogeneidad de las proporciones, y la relación entre el distanciamiento social y la seroprevalencia se evaluó con el modelo de regresión de Poisson (intervalo de confianza de 95% [IC95%]; *P*< 0,05).

**Resultados.:**

De las 4 252 personas que se entrevistaron y a las que se les realizó la prueba, 11,8% (IC95%: 10,8; 12,8) no cumplían el distanciamiento social. La prevalencia de la realización de la prueba rápida con reactivo fue de 4,7% entre quienes no cumplían el distanciamiento social y de 1,9% entre quienes cumplían con esa medida (*P*< 0,05). Las variables relacionadas con el sexo masculino, el grupo etario de 20 a 59 años, la escolaridad de nivel medio, los ingresos familiares mensuales de R$ 3 136,00 a R$ 6 270,00 y la residencia en zonas urbanas guardaron relación con el incumplimiento del distanciamiento social (*P*< 0,05). El cumplimiento con todas las medidas de distanciamiento social fue un factor de protección contra la infección por SARS-CoV-2 (razón de prevalencia: 0,37; IC95%: 0,19; 0,73).

**Conclusiones.:**

Los resultados indican una reducción de la seroprevalencia a causa de las medidas de distanciamiento social.

Desde dezembro de 2019, a síndrome respiratória aguda grave causada pelo coronavírus 2 (SARS-CoV-2) tem demandado a mobilização de instituições internacionais, governantes, profissionais de saúde e populações para o controle e enfrentamento da COVID-19. Em março de 2020, com a declaração de pandemia pela Organização Mundial da Saúde (OMS), diversos países passaram oficialmente a adotar o distanciamento social como uma das estratégias prioritárias de controle da doença.

O distanciamento social visa a reduzir as interações entre as pessoas em comunidades onde os indivíduos infectados, por não terem sido identificados, não foram isolados (1). Tal estratégia é discutida e prevista para esses casos desde 2003, mas somente foi popularizada e adotada em quase todo o mundo a partir de 2020, muitas vezes de forma compulsória, por meio de leis elaboradas em diferentes níveis administrativos (2). O princípio que embasa o distanciamento social se origina nas dinâmicas espaciais que interligam diferentes lugares, desde a escala global até a escala local, considerando a polirritmia das ações sociais (3). Entende-se que os movimentos e encontros entre humanos, animais e diversos produtos, a partir dos diferentes ritmos e velocidades do mundo contemporâneo, aceleram a disseminação de doenças em um mundo interligado.

Refletindo o seu porte continental, o Brasil foi palco de epidemias de COVID-19 diversas, com cidades, estados e regiões apresentando características variadas (4-6). Por exemplo, os resultados do inquérito nacional realizado no primeiro semestre de 2020 demonstraram que, na região Norte, em média, até 25% da população de cidades já haviam contraído a COVID-19; enquanto isso, no Sul, esse percentual se manteve em torno de até 1% (6). Nesse cenário diverso, o distanciamento social, com alteração de diversas práticas, condicionamento das pessoas às suas casas, fechamento de locais e cancelamento de atividades que geram aglomerações (4), trouxe prejuízos ao setor econômico, sendo alvo de críticas e questionamentos por parte da sociedade civil quanto à sua implementação verticalizada pelos governos, que não levaram em consideração as diferenças regionais. No entanto, diante da escassez de testagem em massa e da lentidão no processamento dos testes de transcriptase reversa seguida pela reação em cadeia da polimerase (RT-PCR), que levaram à indisponibilidade de dados significativos sobre a soroprevalência regionalizada da doença, os gestores de saúde investiram no distanciamento como a principal forma de combate à propagação do vírus (7). De fato, uma série temporal de óbitos pela COVID-19 no município e estado de São Paulo verificou resultados positivos com a implementação das estratégias de distanciamento (8).

No estado do Rio Grande do Sul, no Sul do Brasil, os poucos dados epidemiológicos disponíveis no início da pandemia indicavam baixa soroprevalência, porém com tendência de aumento, e alta adesão ao distanciamento social (9). Ao mesmo tempo, não havia dados sobre a associação entre distanciamento social e circulação viral. Diante do exposto, o objetivo do presente estudo foi investigar a soroprevalência de SARS-CoV-2 em 14 municípios que compõem o Consórcio Intermunicipal de Serviços do Vale do Rio Pardo, no Rio Grande do Sul, Brasil, e analisar a associação entre soroprevalência e adesão às medidas de distanciamento social pela população.

## MATERIAIS E MÉTODOS

**FIGURA 1.**
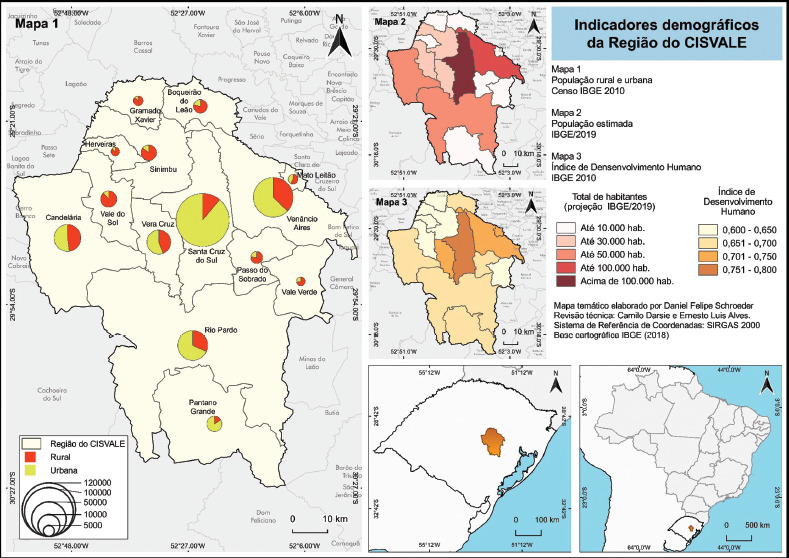
Mapas de indicadores demográficos da região do CISVALE, interior do Rio Grande do Sul, Brasil, 2020^a^

O presente estudo transversal, de base populacional, mensurou a soroprevalência de SARS-CoV-2 na região do Vale do Rio Pardo (COVID-VRP). A região compreende 14 municípios, com população total estimada em 359 mil habitantes (10), dos quais 36,9% residem em área rural (11-14) (figura 1). A pesquisa foi realizada pela Universidade de Santa Cruz do Sul (UNISC) com apoio do Consórcio Intermunicipal de Serviços do Vale do Rio Pardo e das prefeituras e secretarias municipais de saúde.

O cálculo do tamanho da amostra para determinar a soroprevalência de SARS-CoV-2 na região foi realizado usando o *software* Epi Info versão 7 através de amostragem aleatória simples para estimativa de proporções para estudos de base populacional. Foram considerados um nível de confiança de 95%, uma margem de erro de 3 pontos percentuais e um efeito de delineamento igual a 1, totalizando 1 063 indivíduos em cada uma das quatro etapas de coleta de dados realizadas. A amostragem foi distribuída proporcionalmente entre os municípios, de acordo com a população estimada (urbana e rural). As coletas ocorreram nos finais de semana, a fim de garantir uma maior regularidade de indivíduos presentes nas residências.

As três primeiras etapas de coleta de dados sorológicos foram executadas em intervalos de 2 semanas, enquanto a quarta e última etapa ocorreu 5 semanas após a anterior, ou seja: a primeira ocorreu em 1º e 2 de agosto e a última, em 3 e 4 de outubro de 2020 (31ª até 41ª semanas epidemiológicas). O intervalo de 2 semanas foi planejado para possibilitar a verificação da circulação viral, enquanto a última etapa respeitou um período maior para averiguar modificações do perfil da soroprevalência.

O processo de amostragem foi realizado em múltiplos estágios, incluindo amostragem sistemática dos setores censitários, com sorteio de um quarto dos setores censitários em cada um dos 14 municípios. Para a definição do ponto de partida, foi realizado um sorteio simples dos domicílios a serem abordados em cada setor urbano conforme o tamanho de amostra estipulado. Para os setores censitários rurais, foi sorteado apenas o primeiro domicílio a ser visitado. Os demais domicílios foram selecionados a partir dele, incluindo o próximo domicílio na estrada à direita, considerando ambos os lados da estrada.

Os endereços pesquisados em cada setor sorteado foram obtidos a partir do Cadastro Nacional de Endereços para Fins Estatísticos do Instituto Brasileiro de Geografia e Estatística (IBGE) (12). Em cada domicílio, uma pessoa foi sorteada e convidada a participar do estudo. A partir da segunda etapa, a amostragem usou os mesmos setores censitários, mas outros domicílios participantes. Esses domicílios foram selecionados de forma sistemática, a partir da regra do pulo de cinco domicílios à direita, contando desde cada domicílio selecionado na rodada anterior, conforme metodologia já utilizada em estudos prévios (15, 16).

Nas situações em que o domicílio sorteado não correspondia a um endereço residencial, quando não havia ninguém em casa no momento da entrevista ou caso a participação no estudo fosse recusada, o domicílio era substituído pelo próximo domicílio à direita. Para os setores censitários da zona rural, em todas as etapas foi sorteado um novo endereço como ponto de partida para as coletas.

***Fonte:*** GeoSaúde — escala 1:10 (13, 14).

^a^ CISVALE: Consórcio Intermunicipal de Serviços do Vale do Rio Pardo; hab.: habitantes; IBGE: Instituto Brasileiro de Geografia e Estatística.

Na abordagem ao domicílio sorteado, todos os moradores eram listados, mas apenas um era selecionado, também por sorteio, para ser testado. Em caso de recusa, um segundo morador era sorteado. Diante de uma nova recusa, o domicílio era excluído da amostra e os entrevistadores se direcionavam para o próximo domicílio à direita. Igualmente, caso os residentes estivessem ausentes, o próximo domicílio à direita era selecionado.

Todo o procedimento de coleta de dados foi realizado por entrevistadores selecionados entre os profissionais de saúde de nível médio ou superior que atuavam como servidores municipais nas respectivas prefeituras municipais participantes, exceto em um município, onde os entrevistadores foram selecionados entre estudantes dos cursos superiores da área da saúde da UNISC. Cada etapa da coleta contou com a participação de, em média, 110 entrevistadores. Antes de cada etapa, todos recebiam treinamento sobre a realização do teste rápido e as normas de biossegurança, bem como sobre a aplicação do questionário.

Foi utilizado um teste rápido da marca Leccurate® (Lepu Medical Technology, China), que consiste em ensaio imunocromatográfico para detecção rápida e qualitativa de anticorpos IgG e/ou IgM produzidos contra a SARS-CoV-2 em amostras de sangue coletadas por punção digital. O teste é baseado no princípio da reação antígeno-anticorpo a partir da técnica de imunoensaio. O dispositivo de teste contém proteína recombinante SARS-CoV-2 marcada com ouro coloidal, anticorpo IgG humano anticamundongo imobilizado na área de teste G, anticorpo IgM humano anticamundongo imobilizado na área de teste M e anticorpo correspondente na área de controle de qualidade C. Durante o teste, quando o nível de anticorpos SARS-CoV-2 IgM e/ou IgG da amostra está igual ou acima ao limite de detecção do teste, o anticorpo da amostra se liga ao ouro coloidal marcado com SARS-CoV-2. Os conjugados migram através do efeito capilar e são capturados pelo anticorpo IgM e/ou IgG imobilizado posteriormente na área de teste M e/ou G, produzindo uma banda vermelho-púrpura. Em caso de amostra negativa, a faixa vermelho-púrpura não aparece nas áreas de teste M e G. Independentemente da presença ou ausência de anticorpos SARS-CoV-2 na amostra, uma faixa vermelho-púrpura aparecerá na área de controle de qualidade C. A presença da faixa vermelho-púrpura na área de controle de qualidade é um critério para avaliar se há amostra suficiente e se o processo de cromatografia é normal. Conforme informações do fabricante, o teste possui sensibilidade de 77,4% e especificidade de 100% para o IgM. Para o IgG, a sensibilidade é de 96,8%, e a especificidade é de 97,6%.

Para a aplicação do questionário de caracterização da amostra, os entrevistadores utilizaram a versão para dispositivo móvel do Epi Info para coletar variáveis demográficas, socioeconômicas, comportamentais, clínicas e referentes ao uso de serviços de saúde e medicamentos. Foram selecionadas, ainda, três variáveis de desfecho que dizem respeito às medidas comportamentais de distanciamento social, baseadas em estudo anterior. As variáveis “nível de distanciamento social que o entrevistado conseguia praticar”, “rotina de atividades do entrevistado” e “circulação de pessoas na casa” (4) foram medidas pelas seguintes perguntas, cada uma com cinco opções de resposta:

1) Você tem conseguido seguir as orientações de distanciamento social emitidas pelas autoridades de saúde, ou seja, permanecer em casa e evitar contato com outras pessoas? — muito pouco; pouco; mais ou menos; bastante; praticamente isolado.
2) Como tem sido sua rotina de atividades? — fica em casa o tempo todo; sai apenas para coisas essenciais como comprar comida; sai de vez em quando para compras e esticar as pernas; sai todos os dias para alguma atividade; sai todos os dias, o dia todo, para trabalhar ou outra atividade regular.
3) Em relação à rotina domiciliar, quem tem circulado pela casa? — só os familiares que moram junto, se tiver, e mais ninguém; alguns parentes próximos que visitam 1 a 2 vezes na semana; alguns parentes próximos que visitam quase todos os dias; amigos, parentes ou outros que visitam 1 a 2 vezes na semana; amigos, parentes ou outros que visitam quase todos os dias.
  Em seguida, as três variáveis foram dicotomizadas em “sem distanciamento social” e “com distanciamento social”, sendo essa última categoria analisada da seguinte forma: indivíduos que aderiram a uma medida; indivíduos que aderiram a duas medidas; e indivíduos que aderiram a três medidas de distanciamento social. Considerou-se que não realizaram distanciamento social os indivíduos que responderam “pouco/muito pouco” para a questão 1; “saio todos os dias para alguma atividade” e “saio todos os dias, o dia todo, para trabalhar ou outra atividade regular” para a questão 2; e “amigos, parentes ou outros que visitam quase todos os dias” e “alguns parentes próximos que visitam quase todos os dias” para a questão 3. Assim, a variável dependente foi construída, tendo como categoria de referência para análise dos dados os indivíduos que “não realizaram distanciamento social”.

As variáveis independentes avaliadas foram sexo (feminino, masculino), faixa etária (0 a 19, 20 a 59, acima de 60 anos), renda familiar mensal (até R$ 1 045,00, de R$ 1 046,00 a R$3 135,00, de R$ 3 136,00 a R$6 270,00, mais de R$ 6 271,00), escolaridade (analfabeto/não estudou, educação infantil, ensino fundamental incompleto, ensino fundamental completo, ensino médio incompleto, ensino médio completo, ensino superior incompleto, ensino superior completo, pós-graduação), cor da pele autodeclarada (branca, indígena, preta, amarela, parda) e zona de moradia (urbana, rural).

A verificação de consistência e a análise dos dados foram realizadas no *software* STATA 14.0 versão 11. Inicialmente, foi realizada análise estatística descritiva para a caracterização da amostra. Depois, foi realizada análise bivariada por meio do teste do qui-quadrado para heterogeneidade de proporções para variáveis dicotômicas ou nominais e tendência linear para variáveis ordinais.

Para avaliar a associação entre os indivíduos que “não realizaram distanciamento social” e o teste reagente para anticorpos SARS-CoV-2, foi realizado o cálculo das razões de prevalência (RP) bruta e ajustada controladas para sexo, idade, escolaridade e renda, com os respectivos intervalos de confiança de 95% (IC95%), utilizando modelos de regressão de Poisson com variância robusta para estimar as RP.

A pesquisa foi aprovada pelo Comitê de Ética em Pesquisa da UNISC, sob o parecer 4 193 725 (CAAE: 31625220.2.0000.5343). Todos os participantes receberam informações detalhadas sobre os objetivos da pesquisa e os riscos e benefícios de sua participação e assinaram o termo de consentimento livre e esclarecido (TCLE). Para os participantes menores de idade, o TCLE foi assinado por um responsável legal e pela própria criança/adolescente (desde que alfabetizada). O conjunto de dados foi armazenado de forma anônima. Os casos positivos foram notificados aos sistemas de vigilância de COVID-19 municipais.

## RESULTADOS

Foram realizadas análises de 4 252 entrevistas e testes rápidos de SARS-CoV-2. As prevalências de teste rápido reagente nas etapas 1, 2, 3 e 4 foram de 2,9%, 2,2%, 3,3% e 3,4% (*P* = 0,327), respectivamente. A soroprevalência geral de SARS-CoV-2 foi, em média, de 2,9% (IC95%: 2,43; 3,44).

As características da amostra estão apresentadas na tabela 1. Houve maior proporção de mulheres ao longo de todas as etapas (61,0%) e predominância de indivíduos adultos na faixa etária de 20 a 59 anos (58,5%). Em relação à escolaridade, metade dos indivíduos possuía ensino fundamental completo (49,9%) e renda familiar mensal de R$ 1 046,00 a R$ 3 135,00 (49,6%). A cor da pele foi autodeclarada como branca por 86,6% dos participantes. Do total de participantes, 39,4% eram moradores da zona rural.

**FIGURA 2.**
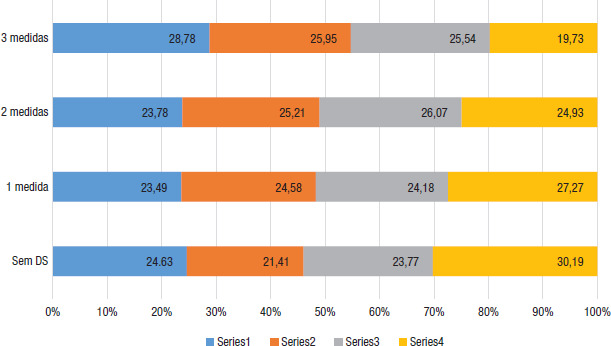
Adesão às medidas de distanciamento social ao longo das quatro etapas do estudo, interior do Rio Grande do Sul, Brasil, agosto a outubro de 2020^a^

^a^ DS: distanciamento social. Series 1, Series 2, Series 3, Series 4 correspondem às etapas do estudo, de 1 a 4, respectivamente. *P* = 0,002 para adesão às medidas de distanciamento social vs. etapas.

A prevalência de indivíduos que não aderiram ao distanciamento social foi de 11,8% (IC95%: 10,83; 12,84) nas quatro etapas. Em todas as etapas, as variáveis sexo masculino, faixa etária de 20 a 59 anos, ensino médio completo e renda familiar mensal de R$ 3 136,00 a R$ 6 270,00 (*P* < 0,05) foram associados à não realização de distanciamento social. Além disso, nas etapas 3 e 4 houve maior prevalência do desfecho de não adesão ao distanciamento social entre os moradores da zona urbana quando comparados aos da zona rural (*P* < 0,05) (tabela 2). Na primeira etapa, verificou-se que 24,6% dos participantes não adotavam medidas de distanciamento social, índice que cresceu para 30,2% na última etapa (*P* < 0,05) (figura 2).

Do ponto de vista dos padrões de distanciamento social, a soroprevalência verificada pelo teste rápido reagente diminuiu com o aumento do número de medidas de distanciamento social praticadas (*P* < 0,001): 4,7% entre aqueles que não cumpriram nenhuma medida de distanciamento social vs. 1,9% entre aqueles que cumpriram todas as medidas de distanciamento social (*P* < 0,05). Observou-se uma redução de 63% do risco de teste rápido reagente para SARS-CoV-2 entre os indivíduos que aderiram às três medidas de distanciamento social quando comparados àqueles que não realizaram distanciamento social (RP: 0,37; IC95%: 0,19; 0,73, *P* < 0,05) (tabela 3). Igualmente, a adesão a duas medidas de distanciamento social demonstrou ser um fator de proteção, com redução de 57% no risco de teste rápido reagente (RP: 0,43; IC95%: 0,26; 0,73, *P* < 0,05).

**TABELA 1.** Descrição da amostra conforme variáveis demográficas, socioeconômicas e resultado do teste rápido para SARS-CoV-2, interior do Rio Grande do Sul, Brasil, agosto a outubro de 2020^a^

**Variáveis**	**Etapa 1**		**Etapa 2**		**Etapa 3**		**Etapa 4**		**Total**	
	**No.**	**%**	**No.**	**%**	**No.**	**%**	**No.**	**%**	**No.**	**%**
Sexo										
Masculino	409	39,6	431	40,6	402	38,0	410	38,6	1652	38,9
Feminino	651	61,4	631	59,4	656	62,0	653	61,4	2591	61,0
Faixa etária										
0 a 19 anos	94	9,0	88	8,4	71	6,7	59	5,6	312	7,4
20 a 59 anos	609	58,6	589	55,8	611	57,7	657	62,0	2466	58,5
≥ 60 anos	337	32,4	378	35,8	377	35,6	344	32,4	1436	34,1
Escolaridade										
Analfabeto/não estudou	30	2,9	34	3,2	26	2,5	29	2,7	119	2,8
Educação infantil	75	7,1	23	2,2	23	2,2	17	1,6	138	3,3
Fundamental	504	48,0	556	52,5	521	49,2	526	49,8	2107	49,9
Ensino médio	269	25,6	268	25,3	303	28,6	266	25,2	1106	26,2
Superior e pós-graduação	172	16,4	178	16,8	186	17,6	219	20,7	755	17,8
Renda familiar (R$)^b^										
Até 1 045,00	168	17,2	174	16,4	208	19,6	164	15,6	714	17,2
1 046,00 até 3 135,00	529	54,4	518	49,0	532	50,0	478	45,4	2057	49,6
3 136 até 6 270,00	207	21,3	211	19,9	187	17,6	211	20,0	816	19,7
Mais de 6 271,00	69	7,1	78	7,4	64	6,0	91	8,6	302	7,3
Não quis informar	-	-	77	7,3	72	6,8	109	10,4	258	6,2
Cor da pele										
Branca	903	87,7	922	86,8	890	83,7	932	88,1	3647	86,6
Não branca	127	12,3	140	13,2	173	16,3	126	11,9	566	13,4
Zona de moradia										
Urbana	704	66,2	616	57,9	630	59,3	626	59,2	2579	60,6
Rural	359	33,8	447	42,0	433	40,7	434	40,8	1673	39,4
Resultado do teste										
Reagente	31	2,9	23	2,2	35	3,3	36	3,4	125	2,9
Não reagente	1032	97,1	1040	97,8	1028	96,7	1027	96,6	4127	97,1

a SARS-CoV-2: síndrome respiratória aguda grave causada pelo coronavírus 2.

b Perda máxima = 108 participantes sem renda familiar.

## DISCUSSÃO

Em contextos como o brasileiro, onde o comportamento da pandemia foi marcadamente diferente em locais distintos, torna-se relevante investigar a pertinência da adesão ao distanciamento social conforme o cenário epidemiológico-sanitário de diferentes regiões e momentos da pandemia, inclusive como forma de obter o apoio da população em relação a essa medida sanitária. O presente estudo evidenciou que a adesão ao distanciamento social foi um fator de proteção contra a disseminação de SARS-CoV-2 em uma região do interior do Rio Grande do Sul. A redução da soroprevalência foi maior entre os indivíduos que aderiram a três medidas de distanciamento social do que entre aqueles que não realizaram distanciamento social. Ou seja, quanto maior a adesão às práticas de distanciamento social, menor a taxa de soroprevalência, denotando um efeito de dose-resposta.

O estudo comprovou, ainda, a baixa soroprevalência de anticorpos contra SARS-CoV-2, conforme ocorria em todo o estado do Rio Grande do Sul no período do estudo (17) e diferentemente de grandes centros do país (18). Destaca-se que, na zona urbana do presente estudo, os entrevistados apresentaram menor frequência de distanciamento social. Pode-se pensar, portanto, que a relação entre zonas urbanas e rurais seguiu o mesmo padrão da interiorização da disseminação da COVID-19, com o distanciamento entre zona urbana e rural retardando a circulação viral (19, 20).

O inquérito EPICOVID-19-BR (5) demonstrou uma so­ro­prevalência no Brasil de 2,9% em maio de 2020. Posteriormente, a média cresceu para 4,6% (4 e 7 de junho de 2020), mantendo-se estável na terceira etapa da pesquisa (21 e 24 de junho). No quarto inquérito, diminuiu para apenas 1,2% (27 e 30 de agosto de 2020). Já no Rio Grande do Sul, um estudo de base populacional (EPICOVID-19-RS) (17) semelhante ao EPICOVID-19-BR apontou uma soroprevalência de 1,22% em agosto de 2020 e de 1,38% em setembro do mesmo ano, porém sem dados regionalizados, apresentando apenas os totais para o estado do Rio Grande do Sul.

**TABELA 2.** Descrição da amostra em relação a adesão às medidas de distanciamento social conforme variáveis demográficas e socioeconômicas, interior do Rio Grande do Sul, Brasil, agosto a outubro de 2020

	Etapa 1			Etapa 2			Etapa 3			Etapa 4			Todas as etapas		
	Distanciamento social			Distanciamento social			Distanciamento social			Distanciamento social			Distanciamento social		
Variáveis	Sem No. (%)	Com No. (%)	*P*	Sem No. (%)	Com No. (%)	*P*	Sem No. (%)	Com No. (%)	*P*	Sem No. (%)	Com No. (%)	*P*	Sem No. (%)	Com No. (%)	*P*
Sexo			0,002^a^			< 0,001^a^			< 0,001^a^			0,013^a^			< 0,001^a^
Masculino	59 (15,8)	315 (84,2)		57 (14,4)	340 (85,6)		58 (15,6)	313 (84,4)		67 (17,5)	315 (82,5)		24 (15,8)	1 283 (84,2)	
Feminino	56 (9,3)	547 (90,7)		43 (7,4)	539 (92,6)		52 (8,4)	571 (91,6)		74 (11,9)	546 (88,1)		225 (9,3)		
Faixa etária (anos)			<0,001^b^			< 0,001^b^			< 0,001^b^			< 0,001^b^			< 0,001^b^
0 a 19	5 (5,8)	81 (94,2)		9 (11,7)	68 (88,3)		13 (18,8)	56 (81,2)		5 (8,8)	52 (91,2)		5 (5,8)	81 (94,2)	
20 a 59	99 (17,7)	461 (82,3)		77 (14,1)	467 (85,8)		87 (15,0)	491 (84,9)		128 (20,4)	499 (79,6)		99 (17,7)	461 (82,3)	
≥ 60	9 (2,9)	302 (97,1)		12 (3,4)	340 (96,6)		11 (3,2)	337 (96,8)		8 (2,5)	307 (97,5)		9 (2,9)	302 (97,1)	
Escolaridade			0,018^b^			0,008^b^			0,003^b^			< 0,001^b^			< 0,001^b^
Analfabeto/não estudou	2 (7,4)	25 (92,6)		1 (3,1)	31 (96,9)		0	26 (100,0)		0	28 (100,0)		3 (2,6)	110 (97,4)	
Educação infantil	6 (8,57)	64 (91,4)		2 (10,0)	18 (90,0)		1 (4,8)	20 (95,2)		2 (11,8)	15 (88,2)		11 (8,6)	117 (91,4)	
Fundamental	45 (9,8)	414 (90,2)		40 (7,7)	476 (92,2)		42 (8,6)	444 (91,4)		48 (9,8)	441 (90,2)		175 (9,0)	1 775 (91,0)	
Ensino médio	39 (15,2)	217 (84,8)		37 (15,2)	207 (84,8)		46 (15,7)	246 (84,2)		44 (17,5)	208 (82,5)		166 (15,9)	878 (84,1)	
Superior/pós-graduação	23 (14,8)	132 (85,2)		20 (12,1)	145 (87,9)		22 (12,8)	150 (87,2)		47 (22,0)	167 (78,0)		112 (15,9)	594 (84,1)	
Renda familiar (R$)^c^			0,126^b^			0,001^b^			0,040^b^			< 0,001^b^			< 0,001^b^
Até 1 045,00	12 (7,8)	141 (92,2)		14 (9,0)	142 (91,0)		14 (7,3)	177 (92,7)		13 (8,3)	143 (91,7)		53 (8,1)	603 (91,92	
1 046,00 a 3 135,00	59 (12,0)	433 (88,0)		39 (8,1)	443 (91,9)		55 (10,9)	451 (89,1)		57 (12,7)	393 (87,3)		210 (10,9)	1 720 (89,1)	
3 136,00 a 6 270,00	29 (14,8)	167 (85,2)		25 (12,7)	172 (87,3)		27 (15,3)	151 (84,8)		40 (20,0)	160 (80,0)		121 (15,7)	650 (84,3)	
Mais de 6 271,00	8 (11,9)	59 (88,1)		16 (22,2)	56 (77,8)		7 (12,1)	51 (89,7)		19 (21,3)	70 (78,6)		50 (17,5)	236 (82,5)	
Cor da pele			0,106^a^			0,885^a^			0,124^a^			0,091^a^			0,564^a^
Branca	92 (11,1)	736 (88,9)		87 (10,3)	761 (89,7)		87 (10,3)	747 (89,6)		130 (14,8)	749 (85,2)		396 (11,7)	2 993 (88,3)	
Não branca	20 (16,1)	104 (83,9)		13 (9,8)	119 (90,2)		24 (14,6)	141 (85,4)		11 (9,1)	110 (90,9)		68 (12,5)	474 (87,45)	
Zona de moradia			0,110^a^			0,082^a^			0,004^a^			0,001^a^			< 0,001^a^
Urbana	85 (12,9)	575 (87,1)		66 (11,6)	501 (88,4)		80 (13,5)	514 (86,5)		102 (17,2)	492 (82,8)		333 (13,8)	2 082 (86,2)	
Rural	30 (9,4)	290 (90,6)		34 (8,2)	379 (91,8)		31 (7,6)	374 (92,4)		39 (9,6)	369 (90,4)		134 (8,7)	1 412 (91,3)	

a Teste do qui-quadrado para heterogeneidade de proporções.

b Teste do qui-quadrado para tendência linear.

c Renda familiar analisada em um amostra de 895 indivíduos na etapa 4.

**TABELA 3.** Associação entre adesão às medidas de distanciamento social e resultado do teste rápido para SARS-CoV-2, interior do Rio Grande do Sul, Brasil, agosto a outubro de 2020 (n = 4 252)^a^

	No. (%)				
Nível de distanciamento social^b^	Não reagente	Reagente	*P*	RP (IC95%) bruta	RP (IC95%) Ajustada^c^
			< 0,001		
Sem distanciamento	445 (95,3)	22 (4,7)		1	1
Uma medida	973 (96,4)	36 (3,6)		0,75 (0,45; 1,27)	0,69 (0,41; 1,16)
Duas medidas	1703 (97,6)	42 (2,4)		0,51 (0,31; 0,85)	0,43 ( 0,26; 0,73)
Três medidas	726 (98,1)	14 (1,9)		0,40 (0,21; 0,77)	0,37 (0,19; 0,73)

a IC95%: intervalo de confiança de 95%; RP: razão de prevalência; SARS-CoV-2: síndrome respiratória aguda grave causada pelo coronavírus 2.

b Medidas de distanciamento social: permanecer em casa e evitar contato com outras pessoas; ficar em casa o tempo todo ou sair apenas para coisas essenciais como comprar comida; circulação de pessoas na casa limitada aos familiares que moram junto.

c RP ajustada controlada para sexo, idade, escolaridade e renda.

Até o momento da pesquisa, ocorreram três fases de desdobramento da soroprevalência de SARS-CoV-2 no Rio Grande do Sul em relação às dinâmicas espaciais. A primeira foi o surgimento da doença, até abril de 2020, em que casos ocorriam na capital, Porto Alegre, e em algumas cidades populosas. A segunda foi o aumento do número de casos registrados pelas capitais regionais com população de 100 a 500 mil habitantes, especialmente em áreas com maiores aglomerados humanos. Por fim, a terceira ocorreu com a difusão do vírus para localidades menores, caracterizando o processo de interiorização da doença no território do estado (21).

Esses resultados corroboram o princípio do distanciamento social, que visa a limitar o contato entre as pessoas e, consequentemente, a contaminação pela doença. Isto é, visa a romper as cadeias de transmissão do vírus, retardar o aumento do número de infectados na comunidade e evitar a sobrecarga dos sistemas de saúde, principalmente dos hospitais.

Muitos países, inclusive o Brasil, implementaram uma série de intervenções destinadas a controlar o número de infectados e desacelerar a velocidade de propagação da doença (4, 22, 23). O governo do Rio Grande do Sul, embora tenha apostado na aplicação do distanciamento social em toda população, sem restrições, conforme o Sistema de Distanciamento Controlado, assim como muitos estados brasileiros (7), inicialmente sofreu várias críticas quanto ao momento mais adequado de implementação. A abordagem do governo estadual foi questionada porque não levou em consideração elementos como a prevalência de SARS-CoV-2 nos diferentes locais, as diferentes dinâmicas de disseminação no interior, a sazonalidade de doenças (como ocorre no inverno na região Sul do Brasil) e as estruturas de atendimento à saúde disponíveis, como leitos de unidades de terapia intensiva e respiradores.

Vale destacar que os dados de soroprevalência deste estudo serviram de suporte técnico para a flexibilização das regras de distanciamento do governo do estado do Rio Grande do Sul, levando em consideração as diferenças regionais (decretos estaduais). Em outras regiões brasileiras, como o Nordeste, o momento da decisão governamental de flexibilização do distanciamento social não atendia aos critérios e parâmetros recomendados pela OMS (24).

Entre as intervenções aplicadas em grande escala para controle da COVID-19, estão o isolamento de indivíduos infectados e seus comunicantes, a higiene das mãos, a etiqueta respiratória e o uso de máscaras em ambientes compartilhados. Além disso, uma série de restrições foram adotadas para garantir o cumprimento do distanciamento social — desde fechamento de escolas e universidades, proibição de grandes festas e assembleias, limitação de viagens e do uso de transporte público sem controle ambiental (fiscalização da higiene de superfícies de uso comum, controle de ar e do espaçamento entre as pessoas), aumentando a consciência pública sobre a necessidade de se permanecer em casa, até a introdução de bloqueio total, com direito de sair apenas para necessidades básicas (25). Em suma, os resultados apresentados sugerem que o distanciamento social observado entre a 31ª e 41ª semana epidemiológica de 2020 colaborou, junto a outras medidas conhecidas, para a manutenção da baixa prevalência de COVID-19 na região do Vale do Rio Pardo (13, 26).

Em relação à idade, os dados seguiram as tendências regionais, visto que 65,52% da população da região é adulta (10, 12). Crianças, adolescentes (população jovem) e idosos apresentaram maior prevalência de distanciamento social, enquanto os adultos de 20 a 59 anos foram os mais expostos, conforme descrito em outros estudos (4, 27). As crianças, devido ao fechamento das escolas e à limitação da realização de outras atividades externas (28), e os idosos, devido à maior mortalidade nessa faixa etária, foram os que mais permaneceram em casa (29). Essa informação foi relevante no momento da realização do estudo, pois havia consenso na literatura de que os idosos e portadores de comorbidades eram os grupos de maior risco para quadros graves e óbitos (27, 30). Atualmente, esse perfil modificou-se devido ao surgimento, em fevereiro de 2021, de uma variante genética da SARS-CoV-2 de preocupação no Brasil (P1) (19, 31, 32).

Em relação ao sexo, as mulheres são culturalmente direcionadas às responsabilidades domésticas e ao cuidado de crianças, enquanto aos homens são projetadas as responsabilidades financeiras do lar (33). No Brasil, a parcela de mulheres responsáveis pelo cuidado da casa é significativamente maior quando comparada à proporção de homens (34). Os dados do presente estudo parecem ir ao encontro dessa realidade, já que as mulheres apresentaram maior prevalência de distanciamento social (90,7%). Ainda, as mulheres representam 50,67% do total da população da região (12), porém contabilizaram 61% da amostra da pesquisa, o que sugere uma maior permanência em ambiente domiciliar.

No presente estudo, observou-se que quanto menor o grau de escolaridade e de renda familiar (até R$ 1 045,00), maior a adesão ao distanciamento social. A hipótese é que os indivíduos com menor escolaridade foram os mais afetados economicamente, o que implica a ponderação de questões relacionadas à perda do emprego, à redução da jornada de trabalho, à diminuição de serviços informais e ao esgotamento de renda para consumo de itens básicos (4, 35, 36).

O tamanho da amostra no presente estudo permitiu uma representatividade da região do Vale do Rio Pardo, incluindo municípios do interior do estado, com zonas urbanas e rurais. Entretanto, uma das limitações do estudo é o potencial viés de memória, uma vez que as informações de distanciamento social foram baseadas em autorrelato. Além disso, o entrevistado pode ter se sentido constrangido em relatar baixa adesão às práticas de distanciamento social. Nesse sentido, o distanciamento social pode estar superestimado. Outra limitação do estudo refere-se ao fato de a soroprevalência ter sido verificada por meio de teste rápido, que apresenta sensibilidade e especificidade variáveis que dependem do tempo de início dos sintomas. Dessa forma, podem ocorrer resultados falsos negativos em pacientes com baixa carga viral e tempo de doença tardio, enquanto falsos positivos podem ocorrer devido à detecção de IgM relacionada a reação cruzada com outra infecção viral. Tal fato não foi controlado, mas não invalida os resultados, conforme verificado em outro estudo (37).

Em resumo, o presente estudo produziu dados robustos e relevantes acerca da soroprevalência de SARS-CoV-2 em uma população do interior do Rio Grande do Sul, inclusive de zona rural, pouco explorada em outras pesquisas conduzidas na primeira onda de COVID-19. Os dados demonstraram que quanto maior a adesão às medidas de distanciamento social, menor a soroprevalência de SARS-CoV-2. Ressaltamos que a colaboração coletiva da população mediante participação em estudos que entregam resultados em tempo real aos governos locais é capaz de amenizar os impactos negativos em casos de emergência de saúde pública.

## Declaração.

As opiniões expressas no manuscrito são de responsabilidade exclusiva dos autores e não refletem necessariamente a opinião ou política da RPSP/PAJPH ou da Organização Pan-Americana da Saúde (OPAS).
